# IMPACT OF THE COVID-19 PANDEMIC ON ELECTIVE KNEE SURGERIES IN ATHLETES

**DOI:** 10.1590/1413-785220243201e275648

**Published:** 2024-05-06

**Authors:** Alexandre Pedro Nicolini, José Manoel Dantas, Orlando Copetti Fração, Vinícius Pagliaro Franco, Alexandre Figueiredo Zobiole, Paulo Vitor Carrijo

**Affiliations:** 1. Universidade Federal de São Paulo, Departamento de Ortopedia e Traumatologia, São Paulo, SP, Brazil.

**Keywords:** Knee Injuries, Anxiety, Athletes, COVID-19, Traumatismos do Joelho, Ansiedade, Atletas, COVID-19

## Abstract

With the COVID-19 pandemic, elective orthopedic surgeries were interrupted in most healthcare services. This leads to impacts on the quality of life, as well as on the emotional, professional, and financial situation of patient athletes who had their surgical treatment postponed. Objective: To evaluate clinical, emotional, and professional impacts on athletes who had their knee surgery postponed. Methods: This study included 21 patients who were diagnosed with knee injuries and were on a surgical waiting list. Participants answered a questionnaire with socioeconomic questions, activity level (amateur/professional), diagnosis, proposed surgery, and questions about anxiety regarding the postponement and uncertainty of performing the surgery, worsening symptoms, and psychological status in general. Results: The most prevalent diagnosis was anterior cruciate ligament injury (81%). Moreover, 42.9% of patients reported being highly anxious about the date of surgery, with 23.8% being highly anxious about the uncertainty of surgery. There was a direct positive correlation (r = 0.418), indicating a higher level of anxiety in patients who faced greater financial impact. Conclusion: The indefinite postponement of surgeries had a great impact on anxiety levels and surgery uncertainty of patients awaiting surgery. ***Level of Evidence III, Transversal Study.***

## RESUMO

*Com a pandemia de COVID-19, as cirurgias ortopédicas eletivas foram interrompidas na maioria dos serviços de saúde. Isso acarretou impacto emocional, profissional, financeiro e na qualidade de vida de atletas que tiveram seu tratamento cirúrgico adiado. Objetivo: Avaliar esse impacto clínico, emocional e profissional em atletas que tiveram suas cirurgias de joelho postergadas. Métodos: Foram selecionados 21 pacientes com diagnóstico de lesão do joelho que estavam aguardando intervenção cirúrgica. Eles responderam um questionário sobre sua situação socioeconômica, nível de atividade (amador/profissional), diagnóstico, cirurgia proposta, ansiedade em relação ao adiamento e incerteza da realização da cirurgia, piora dos sintomas e quadro psicológico de modo geral. Resultados: O diagnóstico mais prevalente foi a lesão do ligamento cruzado anterior (81%). Dos pacientes, 42,9% relataram estar muito ansiosos sobre a data da cirurgia, e 23,8% muito ansiosos quanto à incerteza da cirurgia. Houve correlação positiva direta (r = 0,418) que indicou maior nível de ansiedade nos pacientes conforme o aumento do impacto financeiro. Conclusão: A suspensão indeterminada das cirurgias gerou um grande impacto, com ansiedade e incerteza dos pacientes que estavam aguardando suas cirurgias.* ***Nível de Evidência III, Estudo Transversal.***

## INTRODUCTION

 In March 2020, the World Health Organization (WHO) declared a COVID-19 pandemic due to the rapid spread of the SARS-CoV-2 virus worldwide. The public system and health services were restructured due to the high demand of COVID-19 patients, and as a result, other types of care such as elective surgical procedures were limited and had their appointments postponed. ^1^^,^^2^

 The onset of the COVID-19 pandemic provided unpredictable challenges for the orthopedic community, particularly the subspecialties for knee injuries and sports medicine, as many surgeries were deemed non-essential and, thus, postponed. ^3^

 In this regard, it should be noted that for several elective orthopedic procedures, longer waiting times may negatively affect postoperative outcome. Moreover, some studies have shown that prolonged waiting times are associated with poorer pre-treatment health status. ^4^^,^^5^

 In addition, in these cases, some aspects of mental health often remain underestimated. Psychosocial distress is known to negatively influence postoperative outcomes in patients with musculoskeletal disorders. ^1^

Therefore, the main objective of this study is to evaluate the impact of the COVID-19 pandemic on the quality of life, emotional, professional, and financial situation of athlete patients who had their surgical treatment postponed. As a secondary objective, the correlation between the postponement of elective surgery and weight gain, level of physical exercise during the pandemic, agreement with the maintenance of the proposed treatment, and the patient’s fear of contracting or transmitting COVID-19 during a possible surgical procedure were evaluated.

## MATERIALS AND METHODS

 This is an observational, cross-sectional study conducted at the *Centro Especializado em Trauma do Esporte* (CETE/UNIFESP – Specialized Center for Sports Trauma). All participants signed an informed consent form. This study was approved by the Research Ethics Committee, under opinion no. 5.081.763. This study included adult patients diagnosed with knee injuries who required surgical treatment and were on a surgical waiting list. 

### Selection of participants

A total of 21 patients with knee injuries were selected from the surgical waiting list of the CETE/UNIFESP. After signing the informed consent form, all patients were submitted to a questionnaire with questions about their epidemiological profile: age, sex, sport practiced, level of activity (amateur/professional), diagnosis, and proposed surgery. Moreover, some questions were asked using a Likert scale, regarding anxiety about the postponement and uncertainty of performing the surgery, as well as their psychological status in general.

### Inclusion and exclusion criteria

This study included patients aged > 18 years who signed an informed consent form and had surgical indication for knee injury. Patients aged < 18 years who underwent knee surgery in other services during the pandemic or did not sign the informed consent form were excluded.

### Statistical analysis

 The qualitative characteristics evaluated were described using absolute and relative frequencies for all patients, and the quantitative characteristics were described using means and standard deviations. ^6^ The anxiety questions were described and compared according to the athletic level. For comparisons, the *Mann-Whitney* test was adopted. Spearman’s correlation was calculated to measure the association between the level of anxiety and income impairment. 

The analyses were performed and tabulated using the IBM SPSS for Windows version 22.0 and the Microsoft-Excel 2010 software programs, respectively, considering a 5% significance level.

### Sample estimation

A total of 50 patients were evaluated. After data collection from 25 patients, a sample size estimation was conducted based on the primary objective of the study and the final number of patients was changed to 21 participants.

### Primary outcomes

The postponement of elective surgeries in athlete patients who need surgical treatment impacts on the emotional, quality of life, and professional domains, often making it impossible for these patients to perform their activities. Therefore, there was a significant professional, emotional, and economic impact of the COVID-19 pandemic on athlete patients who require surgical treatment for knee injuries.

### Secondary outcomes

Athletes were deprived of performing their sports activities daily due to the pandemic evolution and the implementation of social isolation measures. The injured athletes had a greater difficulty to maintain physical shape, weight, and physical activity level. They also presented worsening of knee injuries such as pain and instability.

## RESULTS

 A total of 21 patients (2 females and 19 males) were included, with the sample being predominantly composed of males (90.5%). The mean sample age was 27 ± 7.9 years old. At least 57.1% of the sample were amateur athletes, and most performed physical activity five or more times per week (57.1%). Table 1 shows that the most prevalent diagnosis was anterior cruciate ligament injury (81%), followed by meniscus injury (9.5%). 

**Table 1.** Description of personal characteristics and practice of sport and diagnosis for all patients.

**Variable**	**Description (n=21)**
**Sex**	
Female	2 (9.5)
Male	19 (90.5)
**Age (years)**	
Mean ± SD	27 ± 7.9
**Athletic level, n (%)**	
Amateur	12 (57.1)
Professional	9 (42.9)
**Previous frequency of activities, n (%)**	
2× week	1 (4.8)
3× week	3 (14.3)
4× week	5 (23.8)
5× or more per week	12 (57.1)
**Diagnosis, n (%)**	
Anterior cruciate ligament injury	17 (81)
Posterior cruciate ligament injury	1 (4.8)
Meniscus injury	2 (9.5)
Patellofemoral instability	1 (4.8)

Table 2 shows that about 42.9% of the patients reported being highly anxious about the date of surgery. Regarding the uncertainty of surgery, at least 38.1% were moderately anxious and 23.8% were highly anxious. Regarding the pandemic, 42.9% indicated that they were moderately anxious and 23.8% were not anxious. About 76.2% of patients reported that they did not have worsening symptoms during the pandemic. About 23.8% reported being moderately anxious and 23.8% reported being highly anxious about clinical worsening. 

**Table 2.** Description of anxiety and impacts of the injury for all patients.

**Variable**	**Description (n=21)**
**Level of anxiety about date of surgery, n(%)**	
Mildly anxious	5 (23.8)
Moderately anxious	3 (14.3)
Highly anxious	9 (42.9)
Extremely anxious	4 (19)
**Level of anxiety about the uncertainty of performing the surgery, n(%)**	
Not anxious	2 (9.5)
Mildly anxious	3 (14.3)
Moderately anxious	8 (38.1)
Highly anxious	5 (23.8)
Extremely anxious	3 (14.3)
**Pandemic anxiety, n(%)**	
Not anxious	5 (23.8)
Mildly anxious	4 (19)
Moderately anxious	9 (42.9)
Highly anxious	1 (4.8)
Extremely anxious	2 (9.5)
**Agreement regarding the postponement of surgeries, n(%)**	
Total disagreement	2 (9.5)
Mild disagreement	3 (14.3)
Neither agree nor disagree	5 (23.8)
Mild agreement	4 (19)
Total agreement	7 (33.3)
**How determined are you in maintaining the proposed treatment? n (%)**	
I surely want to undergo surgery	19 (90.5)
I am unsure about surgery	2 (9.5)
**How has the postponement impacted you financially? n (%)**	
No impact	6 (28.6)
Low impact	2 (9.5)
Medium impact	6 (28.6)
High impact	3 (14.3)
Very high impact	4 (19)
**Worry / anxiety about returning to sport, n (%)**	
Moderately anxious	4 (19)
Highly anxious	9 (42.9)
Extremely anxious	8 (38.1)
**Worry / anxiety about worsening condition (pain / instability), n (%)**	
Not anxious	3 (14.3)
Mildly anxious	4 (19)
Moderately anxious	5 (23.8)
Highly anxious	5 (23.8)
Extremely anxious	4 (19)
**If surgery is performed, what is your concern about getting COVID 19?, n (%)**	
Not anxious	4 (19)
Mildly anxious	8 (38.1)
Moderately anxious	4 (19)
Highly anxious	2 (9.5)
Extremely anxious	3 (14.3)
**Worsening symptoms during the pandemic, n (%)**	
No	16 (76.2)
Yes	5 (23.8)
**What symptoms?, n (%)**	
Increased pain (I could not undergo physical therapy)	1 (20)
Pain, swelling, and instability	1 (20)
Pain	1 (20)
Lack of sport practice	1 (20)
Total rupture of the ligament, inflammation of other structures of the knee	1 (20)
**Level of physical activity during the postponement period of surgery, n (%)**	
Much smaller than usual	11 (52.4)
Smaller than usual	6 (28.6)
Usual	4 (19)
**Have you gained weight?, n (%)**	
No	14 (66.7)
Yes	7 (33.3)
**If so, how much weight?**	
Mean ± SD	5.4 ± 3.4 kg

 Moreover, at least 52.4% of patients reported a much lower than usual level of physical activity during the pandemic. Only 33.3% reported weight gain, and 38.1% reported being mildly anxious about the risk of contracting COVID-19. At least 28.6% had a moderate impact on income. About 90.5% of the patients intend to maintain the proposed treatment ( Table 2 ). 

Table 3 shows that the level of anxiety for all questions addressed to the patients was statistically similar between amateur and professional athletes (p > 0.05). 

**Table 3.** Description of the anxiety questions according to the athletic level and the results of the comparative tests.

**Variable**	**Athletic level**		**p**
	Amateur (n=12)	Professional (n=9)	
**Level of anxiety about date of surgery**			0.422
Mildly anxious	2 (16.7)	3 (33.3)	
Moderately anxious	2 (16.7)	1 (11.1)	
Highly anxious	5 (41.7)	4 (44.4)	
Extremely anxious	3 (25)	1 (11.1)	
**Level of anxiety about the uncertainty of performing the surgery**			0.808
Not anxious	1 (8.3)	1 (11.1)	
Mildly anxious	2 (16.7)	1 (11.1)	
Moderately anxious	4 (33.3)	4 (44.4)	
Highly anxious	3 (25)	2 (22.2)	
Extremely anxious	2 (16.7)	1 (11.1)	
**Pandemic anxiety**			0.917
Not anxious	3 (25)	2 (22.2)	
Mildly anxious	3 (25)	1 (11.1)	
Moderately anxious	3 (25)	6 (66.7)	
Highly anxious	1 (8.3)	0 (0)	
Extremely anxious	2 (16.7)	0 (0)	
**Worry / anxiety about returning to sport**			0.508
Moderately anxious	3 (25)	1 (11.1)	
Highly anxious	5 (41.7)	4 (44.4)	
Extremely anxious	4 (33.3)	4 (44.4)	
**Worry / anxiety about worsening condition (pain / instability)**			0.754
Not anxious	1 (8.3)	2 (22.2)	
Mildly anxious	3 (25)	1 (11.1)	
Moderately anxious	3 (25)	2 (22.2)	
Highly anxious	2 (16.7)	3 (33.3)	
Extremely anxious	3 (25)	1 (11.1)	
**If surgery is performed, what is your concern about catching COVID 19?**			0.702
Not anxious	2 (16.7)	2 (22.2)	
Mildly anxious	4 (33.3)	4 (44.4)	
Moderately anxious	3 (25)	1 (11.1)	
Highly anxious	2 (16.7)	0 (0)	
Extremely anxious	1 (8.3)	2 (22.2)	
**Level of physical activity during the postponement period of surgery**			0.382
Much smaller than usual	7 (58.3)	4 (44.4)	
Smaller than usual	4 (33.3)	2 (22.2)	
Usual	1 (8.3)	3 (33.3)	
Mann-Whitney test			

Table 4 shows that the pandemic anxiety was statistically equal in patients who did and did not have worsening symptoms during the postponement (p = 0.780). 

**Table 4.** Description of the pandemic anxiety according to worsening symptoms during postponement and comparative test results.

**Pandemic anxiety**	**Worsening symptoms during the pandemic**		**p**
	No	Yes	
Not anxious	4 (25)	1 (20)	
Mildly anxious	3 (18.8)	1 (20)	0.780
Moderately anxious	7 (43.8)	2 (40)	
Highly anxious	1 (6.3)	0 (0)	
Extremely anxious	1 (6.3)	1 (20)	

Table 5 shows that, despite no statistically significant correlation being found between the pandemic anxiety and the degree of financial impact (p = 0.059), the correlation between the two variables was direct (r = 0.418), indicating a higher level of anxiety in patients who reported higher financial impact. 

**Table 5.** Description of the pandemic anxiety according to the financial impact of patients and the result of the correlation.

**Pandemic anxiety**	**How the postponement impacted you financially**					**r (p)**
	No impact	Low impact	Medium impact	High impact	Very high impact	
Not anxious	3 (50)	0 (0)	1 (16.7)	0 (0)	1 (25)	0.418 (0.059)
Mildly anxious	0 (0)	1 (50)	3 (50)	0 (0)	0 (0)	
Moderately anxious	3 (50)	1 (50)	2 (33.3)	2 (66.7)	1 (25)	
Highly anxious	0 (0)	0 (0)	0 (0)	1 (33.3)	0 (0)	
Extremely anxious	0 (0)	0 (0)	0 (0)	0 (0)	2 (50)	

## DISCUSSION

 The COVID-19 pandemic has impacted public health, changing the routine of several sectors. This included the surgical sector, which required postponement of elective surgeries, indicating the need for changes in the way these procedures are conducted. ^7^

 This study found a predominance of males (90.5%) and a mean age of 27 ± 7.9 years. Contrary to these findings, in a study conducted with patients in the waiting list for elective knee replacement during the COVID-19 pandemic, Wilson et al. ^8^ found a predominance of females (64,9%) and a mean age was 65 years. 

 Moreover, in this study, it was observed that most participants (42.9%) were highly anxious about the date of surgery and 38.1% were moderately anxious about the uncertainty of performing the surgical procedure. This corroborates the study by Knebel et al. ^1^ that assessed pre-treatment health status and psychosocial distress after the cancellation of orthopedic surgeries due to COVID-19, finding significant psychosocial distress in some patients due to surgery cancellation. 

 In the study by Dindar ^9^ that investigated the levels of anxiety in the health of handball players during the COVID-19 pandemic, about 40.5% of the participants were professional athletes, differently from our study, in which most patients were amateur athletes (57.1%). However, in this study, there was no statistically significant correlation between the pandemic anxiety, corroborating the study by Dindar ^9^ which also found no significant association between the means of anxiety and COVID-19. 

 Moreover, in this study, the reported levels of anxiety were statistically similar between amateur and professional athletes, similar to the study by Dindar. ^9^

 In this study, about 76.2% of patients reported that their symptoms did not worsen during the pandemic. However, in their study, Wilson et al. ^8^ observed that 23.4% of the patients reported an increase in the dose or frequency of analgesic consumption after being informed about the postponement of surgery and due to worsening symptoms. 

 Similar to the study by Knebel et al., ^1^ about 23.8% of the participants were moderately anxious and 23.8% were highly anxious about clinical worsening. However, 90.5% of the patients intended to maintain the proposed treatment, corroborating the findings of Knebel et al., ^1^ in which the trust of most patients in their surgeons and public system was not affected. 

 In addition, at least 52.4% of patients reported a much lower than usual level of physical activity during the pandemic and only 33.3% reported weight gain. In this context, Sonza et al. ^10^ pointed out that the practice of physical activity was affected during the pandemic, with an increase in sedentary behavior in both active and inactive individuals. Among active individuals, the time spent exercising was significantly reduced, as well as the motivation to exercise, impacting their performance and health during the pandemic. 

 In this study, at least 28.6% had a moderate impact on income. Moreover, a direct positive correlation was found between the pandemic anxiety and the degree of financial impact, indicating that anxiety levels in patients increase according to financial impact. This corroborates the statement by Brooks et al. ^11^ who identified socioeconomic impacts and financial instability as some of the main causes of negative psychological impacts during the pandemic. 

Therefore, we can highlight, as strengths of this study, the categorization of patients by intention. Moreover, this research contributes to better understand the consequences of postponement of elective surgeries on athletes, who are an important niche of society, and the impact of surgical postponement on the quality of life and the professional life of patients.

However, this study presents some limitations. Firstly, the small sample size and the fact that it was restricted to patients who were in the same treatment center. Second, the follow-up period was relatively short; therefore, long-term complications, symptoms, or aggravations could not be reported. Third, the application of questionnaires may reflect some difficulties such as the large number of questions, which may cause demotivation and confusion when answering the questionnaires.

## CONCLUSION

The results of this research indicate that most patients have a reasonable perspective on surgical delays and intend to maintain the proposed treatment. Most patients did not show worsening symptoms during the pandemic. No significant differences were found in anxiety levels between amateur and professional athletes. Higher levels of anxiety were found in patients who faced greater financial impact.

However, the surgical postponement was impactful, as some patients reported being highly anxious to undergo surgery, which can be characterized as emotional distress resulting from the interruption of elective surgery.

While current societal recommendations provide guidance on safety protocols and patient prioritization, each orthopedic practice should consider its unique situation and use evidence-based medicine when determining surgical timing and patient selection.
